# Antibacterial Applications of Nanodiamonds

**DOI:** 10.3390/ijerph13040413

**Published:** 2016-04-12

**Authors:** Sabine Szunerits, Alexandre Barras, Rabah Boukherroub

**Affiliations:** Institute of Electronics, Microelectronics and Nanotechnology (IEMN), UMR-CNRS 8520, University Lille 1, Avenue Poincaré—BP 60069, 59652 Villeneuve d’Ascq Cedex, France; alexandre.barras@iri.univ.lille1.fr

**Keywords:** nanodiamonds, glycans, antimicrobial activity, biofilm inhibition

## Abstract

Bacterial infectious diseases, sharing clinical characteristics such as chronic inflammation and tissue damage, pose a major threat to human health. The steady increase of multidrug-resistant bacteria infections adds up to the current problems modern healthcare is facing. The treatment of bacterial infections with multi-resistant germs is very difficult, as the development of new antimicrobial drugs is hardly catching up with the development of antibiotic resistant pathogens. These and other considerations have generated an increased interest in the development of viable alternatives to antibiotics. A promising strategy is the use of nanomaterials with antibacterial character and of nanostructures displaying anti-adhesive activity against biofilms. Glycan-modified nanodiamonds (NDs) revealed themselves to be of great promise as useful nanostructures for combating microbial infections. This review summarizes the current efforts in the synthesis of glycan-modified ND particles and evaluation of their antibacterial and anti-biofilm activities.

## 1. Introduction

Despite the increased understanding of microbial pathogenesis, interfering with and eradicating the formation of microbial biofilms remain challenging tasks. While the commercialization of penicillin from the late 1940s onwards decreased morbidity and mortality from infectious diseases, the overuse and misuse of antibiotics have led to the emergence of antibiotic resistance in bacterial pathogens. The report entitled *Review on Antimicrobial Resistance* published in December 2014 by O-Neill [1] estimates a death quote attributable to antimicrobial resistance (AMR) of 10 million in 2050 with the costs incurred by drug resistant infections amounting to €1.5 billion annually, due to increases in healthcare expenditure costs. The demand of developing new antimicrobial drugs and therapies for combating bacterial infections has thus become crucial. One strategy intensively investigated in recent years is based on the development of microbiocidal and/or anti-adhesive nanoparticles displaying activity against biofilms [2,3,4,5,6,7]. Various types of nanoparticles such as silver (Ag), silver oxide (Ag_2_O), gold (Au), titanium dioxide (TiO_2_), silicon (Si), copper oxide (CuO), zinc oxide (ZnO), *etc.* have received great attention for their potential antimicrobial activity, inhibiting the growth of several microbial species [8]. Silver nanoparticles (Ag NPs) have been in particular reported to be effective for the destruction of various pathogens and are present in dressings for surgical wounds, in coating for medical devices as well as lotions and gels to prevent bacteria and fungi contaminations. However, concerns about the cytotoxicity of Ag NPs against human cells have been voiced [9]. 

### Glyco-Nanoparticles 

The use of sugar-coated nanoparticles (glyco-nanoparticles) has received sustained attention over the past years [10,11,12,13,14]. Carbohydrates are important components of living organisms and have been identified to play a central role in a large panel of biological processes such as cell-cell communication, viral and bacterial infections, inflammation and immune responses. The extremely low affinity of carbohydrates, typically in the milli- to micromolar range (mM-µM) [15], to biological components is compensated by nature using clustering effects resulting in higher affinities towards the glycan targets. The synthesis of high affinity glycan ligands has been one of the first developed strategies to mimic glycan clustering effects present in nature [14,16,17,18]. Another more recently developed strategy is that of the multivalent presentation of carbohydrate ligands on nanoparticles. This approach has the potential to lead to significantly increased affinities for their appropriate lectin targets compared to monovalent references [19,20,21,22,23,24]. These avidities have shown to be far superior than those arising from a simple additive effect. Indeed, a single particle with a large surface to volume ratio is ready for the attachment of multiple ligands, providing an easy and powerful possibility for enhancing the affinity of glycan-based interactions. 

#### Gold Glycoconjugates

**Figure 1:**
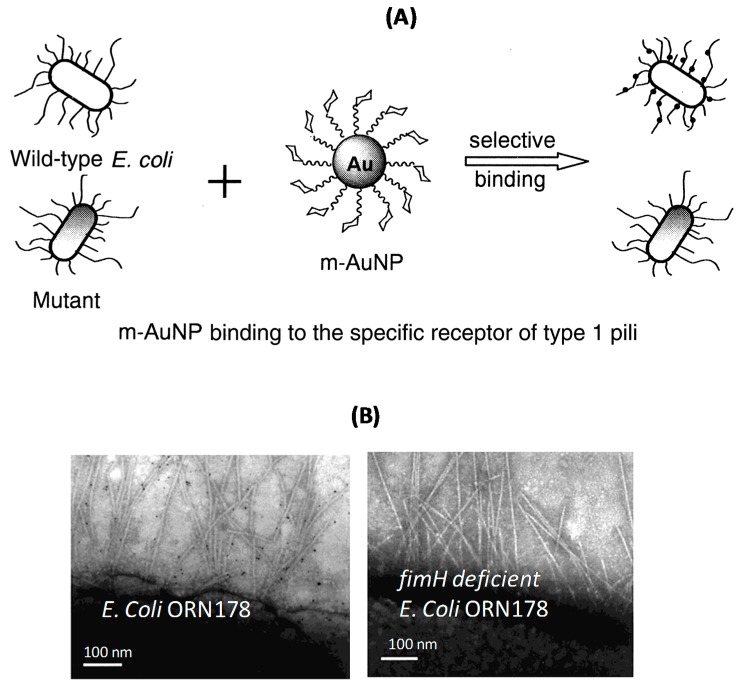

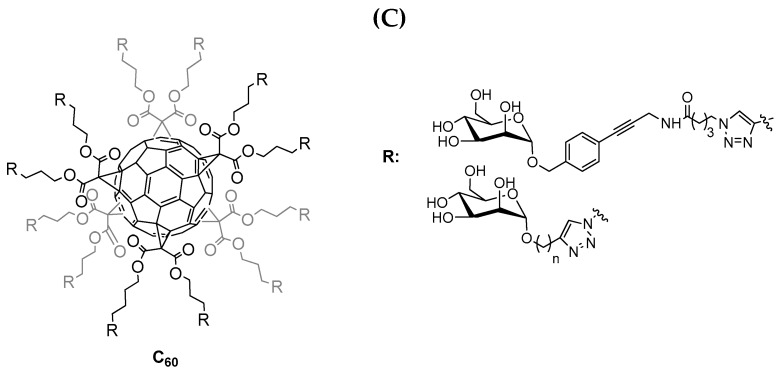
(**A**) Glycan-modified gold nanoparticles (glyco-Au NPs) and their selective binding mechanism to specific receptors of *E. coli* strains; (**B**) TEM images of selected areas of pili of *E. coli* ORN178 strain bound to glyco-Au NPs and fimH gene deficient *E. coli* ORN178 (reprinted with permission from [22]); (**C**) Anti-adhesive glycan-modified fullerenes [19].

One of the first multivalent nano-scaffolds developed and used as biofilm inhibitors are gold glycoconjugates (Figure 1A) [22,25]. The integration of glycan moieties on gold nanoparticles (Au NPs) can be easily achieved through self-assembly of thiolated glycans. Because Au NPs exhibit an intense absorption band (plasmonic band) in the visible region, spectroscopic detection is feasible [26]. The target of these Au NPs based glyconanoparticles are type 1 fimbriae, which constitute major virulence factors produced by *E. coli* [27]. 

Type 1 fimbriae are filamentous tubular structures each of 0.2–2.0 µm in length and 5–7 nm in diameter that are distributed over the entire surface of the bacterium [28]. In various *E. coli* strains, the lectin located at the extremity of type 1 fimbriae, FimH, contributes to tissue colonization through its specific recognition of the terminal α-D-mannopyranosyl units present on cell-surface glycoproteins. FimH-mediated adhesion to such mannosyl moieties is now known to be crucial for the interaction of *E. coli* with uroplakins and consequently for bladder colonization [29]. 

Selective binding of mannose-modified Au NPs to type 1 pili in *E. coli* has proven to be a promising strategy for the development of an anti-adhesive therapy (Figure 1A). Typical TEM images of sectioned areas of pili of *E. coli* ORN178 stain show that gluco-Au NPs cluster around them, while no interaction was observed on the same pathogen deficient of the fimH gene (Figure 1B) [22]. 

#### Fullerene Glycoconjugates

Next to Au NPs, fullerenes have been investigated as glycan scaffolds as they comply well with several requirements needed such stable surface functionalization and good dispersibility in aqueous media (Figure 1C) [16,19]. Fullerene-sugar balls displaying twelve peripheral mannose residues were investigated and compared to the corresponding monovalent model compound. The chemical modification scheme for the conjugation of sugar units to fullerenes was the copper-catalyzed azide-alkyne cycloaddition (CuAAC). These structures were assessed as ligands of the bacterial adhesion FimH. Low nanomolar affinities were measured by isothermal titration calorimetry (ITC) and surface plasmon resonance (SPR). It was also shown that increasing the distance between the mannose residues and the fullerene core improved significantly the binding affinity [18]. Furthermore, the binding profile of galactose-modified fullerenes was investigated against PL-IL (LecA), a bacterial lectin from *Pseudomonas aeruginosa* [16]. Its virulence and binding to host cells are mediated by lectin-carbohydrate interactions involving LecA and LecB, specific for galactose and fructose, respectively. IC_50_ values in the nanomolar range could be again deduced from enzyme-linked lectin assay (ELLA) and SPR measurements, suggesting that fullerene-based glycol clusters are among the most potent anti-adhesive agents against *Pseudomonas aeruginosa* infections. 

#### Glyco-Nanodiamonds

**Figure 2:**
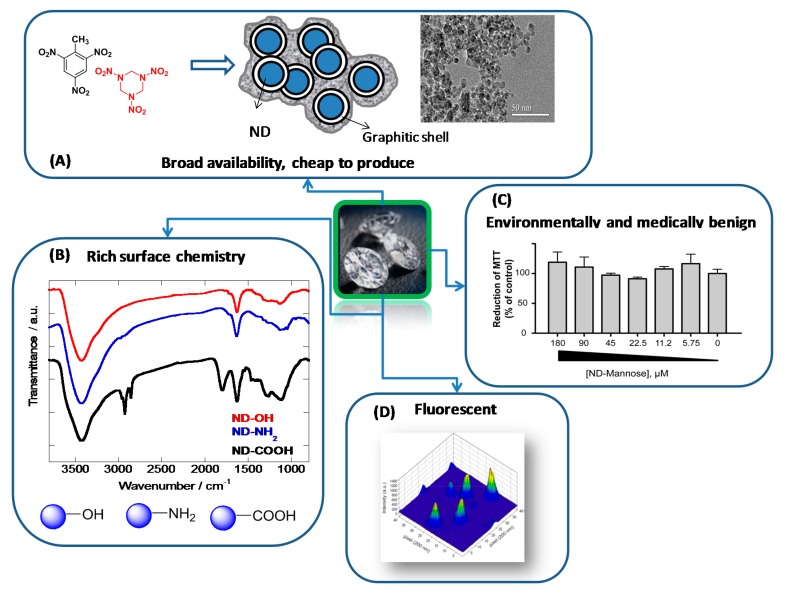
Important aspects of nanodiamonds for biomedical applications: (**A**) Production of detonation NDs from explosives such as trinitrotoluene (TNT) and hexogen (60/40 wt %) in a closed-metallic chamber, which results in diamond-containing soot, and TEM image of hydroxylated ND; (**B**) FT-IR spectra of hydroxylated, aminated and carboxylated NDs [30]; (**C**) Toxicity of mannose modified NDs on T24 bladder cells (reprinted with permission from [10]); (**D**) Confocal scanning image of 35 nm NDs dispersed on a bare glass substrate (reprinted with permission from [42]).

Glycan-modified nanodiamonds (glyco-NDs) have lately been added to the list of nanostructure inhibitors of type 1 fimbriae-mediated *E. coli* adhesion [10,11,12,13,30]. Nanodiamonds (NDs), also named diamond nanoparticles, represent an important class of nanomaterials with outstanding properties (Figure 2). In contrast to metal and metal oxide NPs, NDs are highly stable in corrosive media, thus limiting their decomposition or transformation to materials with potential toxicity and decreased activity. One of the advantages of NDs over other carbon-based materials such as fullerenes is that they are chemically inert, optically transparent, biocompatible, and can be functionalized in many ways depending on their intended ultimate application [31,32,33,34,35,36,37,38,39]. Although their *in vivo* toxicity depends on their particular surface characteristics [40], ND particles do not induce significant cytotoxicity in a variety of cell types [41] and have been used in a variety of biomedical applications. An additional appealing feature of NDs is their intrinsic fluorescence. Diamond crystallites with a nominal size of 100 nm are capable of producing stable fluorescence from color centers after surface treatment with strong oxidative acids. 

These results were not completely unanticipated as the fluorescence of NDs originates from point defects embedded in the crystal lattice, the most noteworthy being the negatively charged nitrogen-vacancy centre (N-V)^−^, which is the dominant end product of thermally-annealed or irradiation-damaged type Ib diamond [42]. These fluorescent NDs are considered non-toxic alternatives to semiconducting quantum dots for biomedical imaging [33,42]. A variety of methods are currently available for the fabrication of non-fluorescent or fluorescent NDs. Next to ball milling of synthetic or natural diamond microcrystals [43], laser ablation [44], irradiation of graphite [45] or chlorination of carbides [46], the use of detonation techniques [47,48] is still the most widely used technique. It is based on the production of NDs from explosives such as trinitrotoluene (TNT) and hexogen (60/40 wt %) in a closed-metallic chamber, which results in diamond-containing soot (Figure 2). While the particle size of its primary crystallites is ~5 nm with a very narrow size distribution [49,50], due to harsh conditions in the reaction chamber, detonated ND particles exist mainly in the form of strongly bound agglomerates [51]. The particles are not only linked by the usual electrostatic interactions by also via covalent bonds between surface functional groups as well as by soot structures surrounding each primary particle [52,53]. The detonation soot can be removed using oxidizing mineral acids (HNO_3_, mixtures of H_2_SO_4_ and HNO_3_, K_2_Cr_2_O_7_ in H_2_SO_4_, KOH/KNO_3_, HNO_3_/H_2_O_2_ under pressure, *etc.*) [54], as the reactivity of disordered sp^2^ carbon is higher than that of diamond, thus removing non-diamond impurities. During the cleaning step, the surface of NDs is covered with a variety of functional groups such as hydroxyl, carbonyl, carboxyl, anhydrides and lactones (Figure 2), allowing a wide range of surface functionalization strategies to be implemented. 

The properties listed in Figure 2 have been determinant in the choice of NDs to explore their utility to combat viral [55] and bacterial infections [10,11,13,27,56,57]. While there are only a handful of papers on the antibacterial properties of ND particles as well as their ability to interfere in biofilm formation, the results of these studies are highly encouraging. The purpose of this short review is to discuss some of these findings and give a general outline on the advances in this field. 

## 2. Design of Glyco-Nanodiamonds (Glyco-NDs)

**Figure 3:**
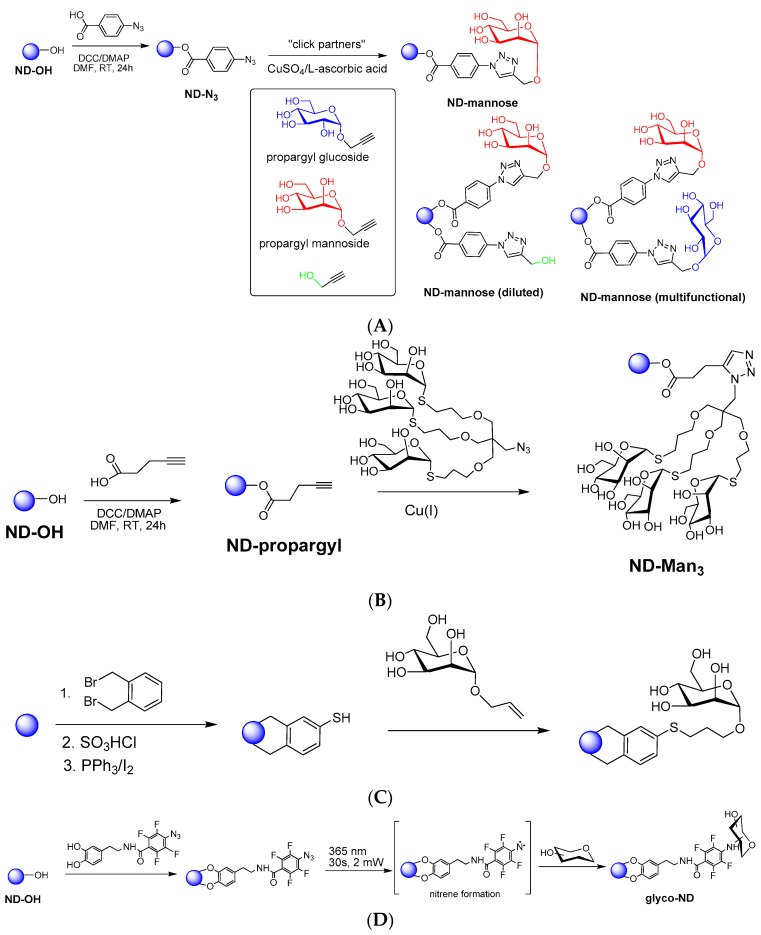
Different surface functionalization strategies for the formation of glycan-NDs: (**A**,**B**) copper-catalyzed azide-alkyne cyloaddition (CuAAC) [10,13]; (**C**) Thiol-ene reaction [12]; (**D**) photo-induced covalent attachment of native carbohydrates [59].

Despite their evident potential for glycobiology, there are only a few reports on the design of glyco-nanodiamonds (glyco-NDs) (Figure 3). One class of surface functionalization routes that is very attractive for the quick, simple and efficient grafting of organic moieties is the so-called “click” chemistry. This strategy was exploited for the construction of one of the first generation of glyco-NDs through the covalent conjugation of propargyl-terminated sugar moieties to azide-functionalized NDs (Figure 3A) [10]. The azide termination was obtained through esterification reaction of the surface hydroxyl groups of commercially available ND-OH particles with 4-azidobenzoic acid in the presence of *N*,*N*’-dicyclohexylcarbodiimide and a catalytic amount of 4-dimethylaminopyridine. The fabricated azide-functionalized ND particles (ND-N_3_) reacted smoothly with propargylated partners in the presence of CuSO_4_/L-ascorbic acid as catalyst giving the corresponding modified particles. The interest of this strategy lies in addition in the possibility to form diluted and multifunctional particles, opening widely the possibilities of interaction with different pathogens. The second generation of glyco-ND was obtained through reaction of ND-OH with 4-pentynoic acid using *N*,*N*’-dicyclohexyl-carbodiimide and a catalytic amount of 4-dimethylaminopyridine (DMAP) to give the corresponding ND-propargyl (Figure 3B). The propargyl groups thus installed on the NDs surface were then reacted with appropriate azido-derivatized partners, trithioglycans in this case, in the presence of CuSO_4_/ascorbic acid as catalyst to give the corresponding sugar cluster-clicked NDs [13]. 

Krueger and co-workers used a multistep reaction including a Diels-Alder cycloaddition of 1,2-dimethylbromide phenol to the NDs surface, followed by a classical aromatic sulfonation and reduction to thiol (Figure 3C) [12]. The thiol-terminated NDs were used as anchors for allyl modified glycans in a “thiol-ene” type reaction to give glycan-modified NDs.

More recently, the interest of dopamine and its derivatives for the direct functionalization of NDs has been demonstrated [31,58]. Dopamine, chemically known as 4-(2-aminoethyl)-benzene-1,2-diol, has sparked great interest as an anchor for the functionalization of metal oxide surfaces because of the stability and strength of the resulting five-membered metallocyle chelate. Hydroxylated NDs can be directly functionalized *via* the hydroxyl groups with dopamine anchors bearing different functions such as perfluoroarylazide groups (Figure 3D). Taking advantage of the photochemistry of arylazides that can be easily converted to reactive nitrenes upon light activation, the covalent attachment of native carbohydrates is feasible [59]. 

The resulting highly reactive nitrene intermediates are believed to interact with glycans through C-H and/or N-H insertion reactions, creating highly robust covalent linkages. We have recently compared the effectiveness for lectin-recognition ability of mannose-modified surfaces formed photochemically or *via* Cu(I)-catalyzed “click” chemistry using surface plasmon resonance (SPR) [60]. Although photochemical surface conjugation did not give surface attachment specifically through anomeric center, this was seen not to have a bearing on the SPR behavior of the fabricated sugar interfaces, maintaining their expected binding affinity and specificity towards their partner lectins.

## 3. Application of Glycol-NDs for Combating Bacterial Infections

### 3.1. Antimicrobial Activity 

**Figure 4:**
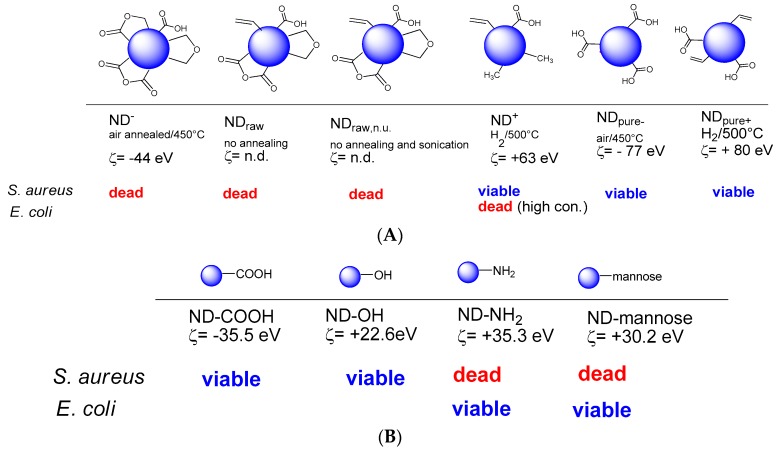
Antibacterial effect of different NDs: (**A**) Antibacterial activity of differently oxidized NDs on *E. coli* (a) and *B. subtilis* (b) evaluated using the adenosine triphosphate (ATP) level as marker for vital bacterial metabolism [57]; (**B**) Antibacterial activity of ND-COOH, ND-NH_2_, ND-OH and ND-mannose determined from fluorescence images using Dead/Live assays [30].

Before the use of NDs for interfering with the formation of microbial biofilms, some of the first studies related to NDs concerned their antibacterial activity. Beranova *et al.* investigated for example the effect of as-synthesized ND particles on the growth of Gram-negative *E. coli* and found that the presence of NDs on agar plates significantly reduced the colony formation ability of *E. coli* [61]. The antibacterial effect occurred in a concentration dependent, but non-linear manner. A NDs concentration of 5 µg/mL gave rise to a 25% inhibition of number of colonies on LB agar plates, while a 100% growth inhibition could only be reached with concentrations above 50 µg/mL [61]. It was hypothesized that NDs clustering around bacterial cells results in blocking essential cellular functions, being responsible for the antibacterial action of NDs. More recently, a comprehensive study on the importance of surface chemical termination on the antimicrobial properties of NDs was conducted by Wheling and colleagues (Figure 4A) [57]. While negatively charged NDs showed a strong antibacterial activity against *E. coli* and *B. subtilis*, positively charged ones caused bacterial death only at high ND concentrations. The antibacterial activity of the ND particles was suggested to be linked to the presence of partially oxidized and negatively charged surface functions, and more specifically to acid anhydride groups [57]. 

In order to shed more light on the influence of surface composition and charge on the NDs antimicrobial properties, the antibacterial activity of carboxylated NDs (ND-COOH), aminated NDs (ND-NH_2_) and hydroxylated NDs (ND-OH) was evaluated recently by us against Gram-positive (*S. aureus* NCTC 6571) and Gram-negative *E. coli* (strain NCTC 8196) bacteria. While ND-COOH and ND-OH particles did not exhibit antibacterial activity, the ND-NH_2_ particles showed bactericidal activity for *S. aureus*, but not for *E. coli* (strain NCTC 8196) (Figure 4B). The contrasting behavior of ND-NH_2_ against *S. aureus* *vs.* *E. coli* might be due to the interaction of the surface NH_2_ groups with the peptidoglycan layer of Gram-positive *S. aureus* and perhaps to interference of ND-NH_2_ with bacterial binary fission. By the same token, ND-NH_2_ would not be expected to interact physically with the exterior lipid membrane of Gram-negative *E. coli*, which features an outer phospholipid membrane serving to shield its internal peptidoglycan layer and is thus quite different from that of *S. aureus.* In addition to these ND particles, the antibacterial character of mannose-NDs was also looked at in a similar manner. An unpredicted bactericidal activity of mannose-modified NDs for *S. aureus* was found, while the same particles exhibited no effect on *E. coli* (Figure 4B).

### 3.2. Inhibition of Biofilm Formation

Next to the bactericidal effect of the different ND structures, their potential for interfering with the formation of microbial biofilm has been investigated. Despite the increased understanding of microbial pathogenesis [62], interfering with the formation of microbial biofilms and disrupting the established ones remain a challenge. Bacteria residing within a structured biofilm community behave markedly differently from those that are free. As a consequence, the development of new antimicrobial agents and identification of the factors that lead to biofilm growth inhibition, biofilm structure disruption or eradication of biofilm formation is a pressing goal. 

**Figure 5:**
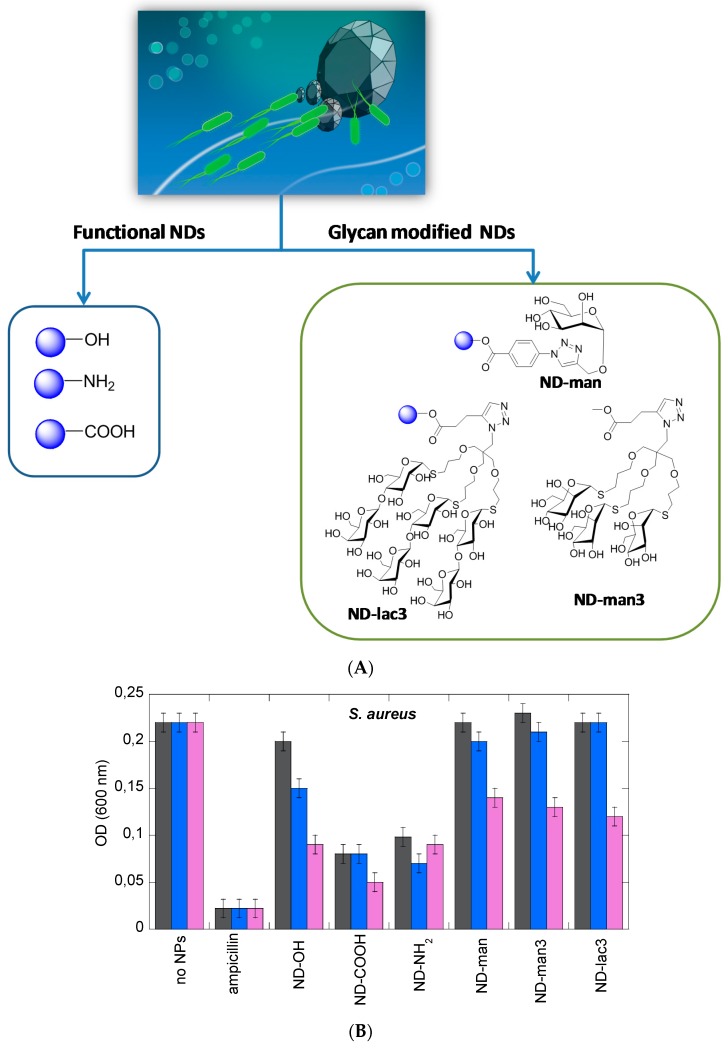

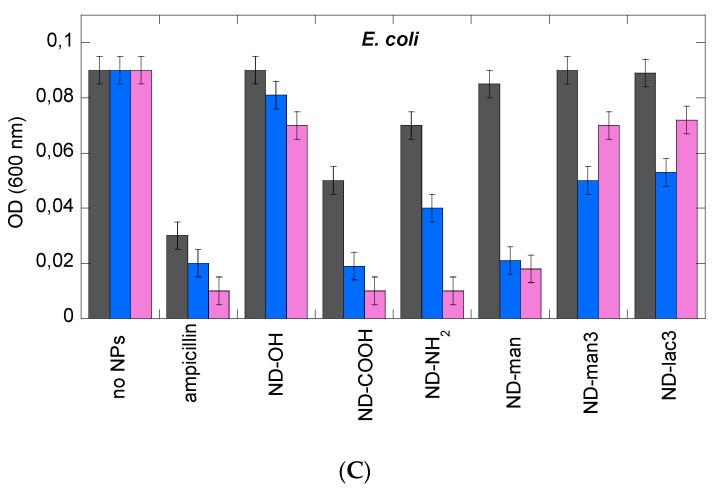
(**A**) Structures of the different ND particles investigated against biofilm formation; (**B**,**C**) Determination of the effect of ND particles on the inhibition of biofilms by mixing overnight cultured *S. aureus* (**B**) and *E. coli* (**C**) suspensions with different concentrations (1 μg/mL, black; 10 μg/mL, blue; 50 μg/mL, pink) of ND particles and further incubation at 37 °C for 24 h with no agitation in 96-well agar plates. Wells were washed to remove non-adherent cells and biofilms were stained by adding crystal violet. The bacterial cell density was determined by measuring the optical density in each well at 600 nm (OD_600 nm_) [30].

**Figure 6:**
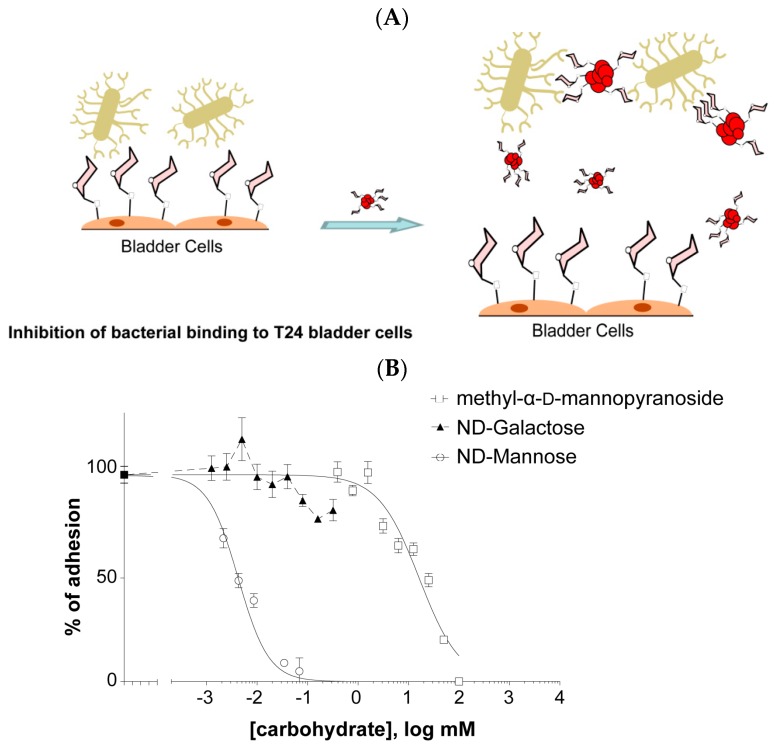

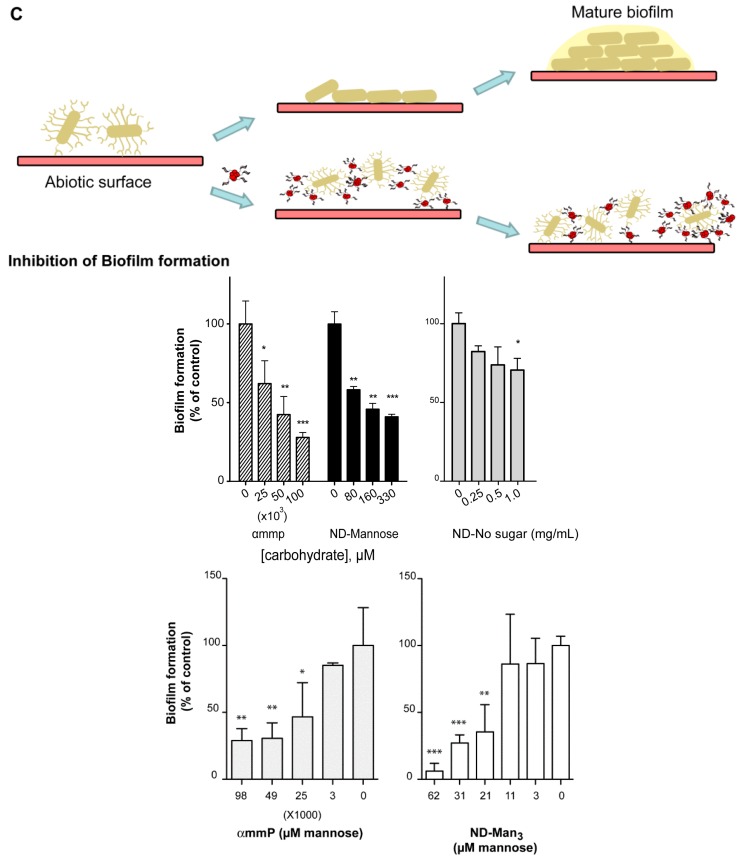
Schematic representation of the ability of ND-man to counteract FimH-mediated adhesion: (**A**) ND-man inhibits binding of type 1 fimbriated cells to T24 epithelial bladder cells. Bacteria were incubated with varying concentrations of each compound to be tested and added to a confluent T24 cells monolayer on 96 well plates. After washing, the attached bacteria were measured by fluorescence in an Infinity 200 (Tecan) plate reader and expressed as relative fluorescence units (R.F.U.). Data are expressed as percentage of adhesion of bacteria with respect to that in the absence of drug. Increasing amounts of αmmp or NDs significantly reduce the binding of bacteria to cells (reprinted with permission from [10]); (**B**) Inhibitory effects of methyl-α-D-mannopyranoside and NDs on biofilm formation. The various compounds were individually added at the start of biofilm growth within microtiter plates. After 24 h of growth, biofilm formation was evaluated using crystal violet staining. (**C**) The biofilm formation of type 1 fimbriated strain was reduced in the presence of αmmp, ND-man and ND-man3 while no or reduced inhibition was observed in the presence of ND-No Sugar. Data is expressed as percentage of growth in the absence of drug. Bars represents mean ± SD, *n* = 3. * *p* < 0.05, ** *p* < 0.01, *** *p* < 0.001 (reprinted with permission from [10,13]).

Anti-adhesive nanoparticles displaying activity against biofilms have recently been developed, among them NDs [10,12,13,56]. The majority of ND particles investigated (Figure 5A) were found to inhibit *S. aureus* induced biofilm formation in a dose-dependent manner (Figure 5B). However, the levels of inhibition are rather moderate with the exception of ND-NH_2_, ND-COOH and ND-OH particles for which good biofilm disruption was observed at the highest particle concentrations tested. The data for *S. aureus* is in striking contrast to that for *E. coli* (Figure 5C). High levels of biofilm inhibition are observed for *E. coli* and in particular with ND-NH_2_ and ND-COOH NPs. At concentrations of 50 µg·mL^−1^, the levels of inhibition attained with these particles are comparable with that obtained with ampicillin. Mannose-modified NDs were also extremely efficient to interfere with biofilm formation of uropathogenic *E. coli* [10,13,30]. In the case of uropathogenic *E. coli* UTI89, FimH contributes specifically to bladder colonization through binding to terminal α-D-mannosyl units present on glycoproteins such as uroplakins. This major virulence factor of *E. coli* is located at the tip of type 1 fimbriae, consisting of filamentous tubular structures (0.2–2.0 µm in length and 5–7 nm in diameter), distributed over the entire surface of the bacterium. The affinity of an individual FimH protein receptor for mannose or high-mannose glycans and even specifically designed synthetic monovalent mannoside-derived ligands is often disappointingly low. The use of mannose-modified NDs has shown to be able to overcome some of these limitations through the demonstration that ND-man and ND-man3 (Figure 5A) effectively inhibit type 1 fimbriae-mediated yeast-agglutination and human bladder-cell adherence in a sugar-selective manner (Figure 6). The eukaryotic cell-adherence inhibitory efficiency of these particles revealed to be far superior to those reported for other glycan-modified particles and nanostructures directed against *E. coli*. In addition, these particles display an *E. coli* biofilm inhibitory activity, a property not previously reported for multivalent glyco-nanoparticles (glyco-NPs), and rarely for other sugar analogs specifically targeting FimH [63,64].

## 4. Conclusions 

Despite the fact that we live in an era of advanced technologies for elucidating underlying mechanisms of infectious diseases and designing new anti-bacterial drugs, infections continue to be one of the greatest health challenges worldwide. Diamond nanoparticles have been used more intensively in recent years in combating infectious diseases and biofilm formation and have proven to be a viable alternative to common antibiotics. Mannose modified-NDs have in particular shown high potential in countering *E. coli-*biofilm formation. While too early to advance further, it might be expected that these nanostructures have advantages over either bactericidal or bacteriostatic therapies, in being less prone to encourage resistant strains. Further evaluation of these structures as potential anti-adhesives for countering bacterial colonization and infection *in vivo* is urgently needed to be able to value the full antibacterial properties of ND particles. While there is still a long way to go, it seems that the future of ND particles to fight bacterial infections is very promising.
